# Automatic Detection and Quantitative DCE-MRI Scoring of Prostate Cancer Aggressiveness

**DOI:** 10.3389/fonc.2017.00259

**Published:** 2017-11-10

**Authors:** Nestor Andres Parra, Alan Pollack, Felix M. Chinea, Matthew C. Abramowitz, Brian Marples, Felipe Munera, Rosa Castillo, Oleksandr N. Kryvenko, Sanoj Punnen, Radka Stoyanova

**Affiliations:** ^1^Department of Radiation Oncology, University of Miami Miller School of Medicine, Miami, FL, United States; ^2^Department of Radiology, University of Miami Miller School of Medicine, Miami, FL, United States; ^3^Department of Pathology, University of Miami Miller School of Medicine, Miami, FL, United States; ^4^Department of Urology, University of Miami Miller School of Medicine, Miami, FL, United States

**Keywords:** prostate cancer, DCE-MRI, image processing, pattern recognition, mpMRI

## Abstract

**Purpose:**

To develop a robust and clinically applicable automated method for analyzing Dynamic Contrast Enhanced (DCE-) MRI of the prostate as a guide for targeted biopsies and treatments.

**Materials and methods:**

An unsupervised pattern recognition (PR) method was used to analyze prostate DCE-MRI from 71 sequential radiotherapy patients. Identified regions of interest (ROIs) with increased perfusion were assigned either to the peripheral (PZ) or transition zone (TZ). Six quantitative features, associated with the washin and washout part of the weighted average DCE curve from the ROI, were calculated. The associations between the assigned DCE-scores and Gleason Score (GS) were investigated. A heatmap of tumor aggressiveness covering the entire prostate was generated and validated with histopathology from MRI-ultrasound fused (MRI-US) targeted biopsies.

**Results:**

The volumes of the PR-identified ROI’s were significantly correlated with the highest GS from the biopsy session for each patient. Following normalization (and only after normalization) with gluteus maximus muscle’s DCE signal, the quantitative features in PZ were significantly correlated with GS. These correlations straightened in subset of patients with available MRI-US biopsies when GS from the individual biopsies were used. Area under the receiver operating characteristics curve for discrimination between indolent vs aggressive cancer for the significant quantitative features reached 0.88–0.95. When DCE-scores were calculated in normal appearing tissues, the features were highly discriminative for cancer vs no cancer both in PZ and TZ. The generated heatmap of tumor aggressiveness coincided with the location and GS of the MRI-US biopsies.

**Conclusion:**

A quantitative approach for DCE-MRI analysis was developed. The resultant map of aggressiveness correlated well with tumor location and GS and is applicable for integration in radiotherapy/radiology imaging software for clinical translation.

## Introduction

Prostate cancer is the second most common cancer in American men (1). Clinical decisions, related to the need for prostate biopsy, define target areas for biopsy and regions that require attention in focally directed therapy, are multifactorial and complex. Delivery of targeted radiation dose to high-risk tumor areas in lieu of escalating dose to the entire prostate will reduce overall complication risks (2), a strategy that requires robust and quantitative imaging of potential tumor lesions (3, 4).

The use of multiparametric MRI (mpMRI) for prostate cancer is rapidly evolving because of its growing availability and ability to combine functional [perfusion *via* dynamic contrast enhanced (DCE-MRI) and diffusion *via* diffusion-weighted imaging (DWI)], and anatomical information [T2-weighted (T2w) MRI]. DCE-MRI is an established component of prostate mpMRI, and DCE-MRI alone has reported sensitivity and specificity ranges of 46–96 and 74–96%, respectively (5–10). The role of DCE-MRI for tumor diagnosis and assessment in the current version of Prostate Imaging Reporting and Data System (PI-RADS) (version 2), however, is minimized (11). DCE-MRI is only considered when DWI in the peripheral zone (PZ) is indeterminate. The PI-RADS Steering Committee justified the reduced role of DCE-MRI in prostate cancer assessment by the lack of expert consensus, reflective of the difficulty of interpreting DCE-MRI sequences by eye. An automated and quantitative assessment has the potential to improve consistency in identifying high risk prostate volumes.

There has been considerable efforts to standardize the analysis of DCE-MRI (12, 13) with pharmacokinetic modeling (14); however, there is significant variability in the calculated rate constants. For instance, K^trans^, the volume transfer coefficient that measures capillary permeability (14), calculated on the same DCE-MRI data by participants from several academic medical centers yielded a within-subject coefficient of variation of 0.59 (15). This modeling approach is impeded by difficulties in estimating the arterial input function (16), insufficient temporal resolution (17, 18) and low signal-to-noise ratio (SNR).

Previously, it has been shown that the application of pattern recognition (PR) techniques to DCE-MRI overcomes the challenges of low temporal resolution and low SNR (19–21). Here, the PR approach is extended by introducing novel quantitative features that allow for comparisons between patients. The method automatically: (i) delineates the region in the prostate with increased perfusion; (ii) allocates the dominant lesion to either the PZ or transition zone (TZ); (iii) assigns a DCE score—a zone-specific quantitative measure of aggressiveness; and (iv) generates a spatial map of tumor aggressiveness based on DCE score. The result is a color-coded map of aggressiveness on a pixel level, which may serve as a guide for targeted biopsies and treatments.

## Materials and Methods

### Patients and MRI Acquisition

An Institutional Review Board (IRB) approved a protocol for retrospective review of mpMRI exams from prostate cancer patients. The IRB waived the need for informed consent. A total of 71 sequential patients, presenting for evaluation for radiation treatment (RT) between 2012 and 2015 and who underwent mpMRI exams on a 3T Discovery MR750 (GE, Waukesha, WI, USA) were identified from the departmental database.

mpMRI exam consisted in part of: (i) axial T2w-MRI of the pelvis: resolution 1.25 mm × 1.25 mm × 2.5 mm; field of view: 320 mm × 320 mm; slice thickness = 2.5 mm (no gap); 72 slices, and (ii) DCE-MRI—12 series of T1 weighted (T1w) at 30–34 s temporal resolution, TR = 3.77–4.05 ms, TE = 1.69–1.78 ms acquired following intravenous bolus injection of a paramagnetic gadolinium chelate—0.1 mmol of gadobenate-glumine (Bracco Diagnostics Inc., Princeton, NJ, USA) per kilogram of body weight. The contrast is administered with a power injector (Spectris, Medrad Inc., Warrendale, PA, USA) at 2 mL/s and followed by a 20-mL saline flush.

### Quantitative DCE-MRI Analysis

**Figure 1:**
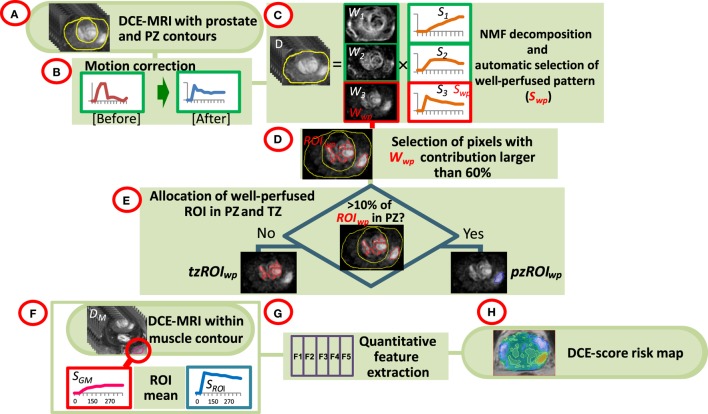
Workflow of quantitative DCE-MRI analysis. **(A)** The input consists of DCE-MRI and contours of the prostate, peripheral zone (PZ), and a sample of gluteus maximum (GM); **(B)** Motion correction of the prostate; **(C)** Non negative matrix factorization (NMF). The data are presented as a product of three temporal patterns (*S*_1_, *S*_2_, *S*_3_) and their magnitudes (*W*_1_, *W*_2_, *W*_3_). The well perfused pattern *S*_wp_ is identified between the three patterns as the pattern with the largest AUC for the first 90 s; its corresponding weights *W*_wp_ represent an intensity map of the distribution of the well-perfused pixels in the data; **(D)** *W*_wp_ is segmented to identify the suspected for tumor region of interest *ROI*_wp_; **(E)** *ROI*_wp_ is assigned to PZ if >10% of *ROI*_wp_ is within the PZ contour and vice versa; **(F,G)** Series of quantitative features (DCE-score) are computed using the signal-vs-time *S*_ROI_ from *ROI*_wp_ and *S*_GM_ of GM; **(H)** A spatial map of tumor aggressiveness is computed using DCE-score and *W*_wp_.

The analysis pipeline is presented schematically in Figure 1. The DCE-MRI series were uploaded in MIM (MIM, Cleveland, OH, USA) and all subsequent analyses are performed using MATLAB R2014a (MathWorks, Natick, MA, USA) plugins. Prostate, PZ, and sample gluteus maximus (GM) volumes were manually contoured (Figure 1A). The individual steps of the analysis are described below:

- *Motion correction* (Figure 1B). Each imaging set in the DCE-MRI series was aligned to the preceding one by finding the affine transformation (shifting, scaling, and rotation) that maximized their mutual information (22). Pixels within the prostate contour and extension by 18.75 mm (15 pixels) were considered. For each pair of before and after-correction images, the sum of squared pixel differences was computed and motion correction was carried out only when this sum was smaller post-correction.
- *Identification of well-perfused (suspicious) regions of interest (ROI) in the prostate*. Non negative matrix factorization (NMF) was applied to signal-vs-time curves of all pixels within the prostate (Figure 1C) (19). Briefly, if *D* is the data matrix, containing the individual pixel’s signal-vs-time curves in its rows (baseline corrected by the average of the pre-contrast points), then *D* can be represented as a product of *k* basic temporal contrast signatures *S*(*t*) and their weights *W*(*X*) in each pixel, i.e., *D* ~ *W* × *S* under the constraint that all elements of *W* and *S* are non-negative. *k* = *3* is estimated by Principal Component Analysis of *D* as the number of significant Principal Components (23). The signal in the *i*^th^ pixel in data matrix D can be represented as:
  (1) $$D_{i}\sim W_{1,i}\times S_{1}+W_{2,i}\times S_{2}+\ldots +W_{k,i}\times S_{k}$$
  The well-perfused pattern, *S*_j_, is automatically selected from the *k* NMF patterns as:
  (2) $$S_{j}=\text{max}\left({\text{AUC}}_{0-90}\left(S_{m}\right)\right),m=1,\ldots ,k$$
  where AUC_t1-t2_ (*S*_i_) is the area under the curve (AUC) of the pattern *S*_m_ between times *t1* and *t2*. Let *W*_j_ be the well-perfused (wp) contribution map (*W*_wp_) associated with the well-perfused pattern *S*_j_ (*S*_wp_). *W*_wp_ is an intensity map of the “well-perfused” pixels in the data (Figure 1D).
- *Segmentation of well-perfused ROI*. Five methods to segment *W*_wp_ were tested for identification of the *ROI*_wp_, the well-perfused ROI. The first was based on Otsu thresholding (24); the others were based on a parameter β = 40, 50, 60, and 70, describing how “pure” is the pattern in a pixel (20). For instance, if β = 60, the *ROI*_wp_ is defined by pixels for which the weight of the well-perfused pattern *W*_wp_ is >60% of the total sum of the weights (Eq. 1). Simultaneously, the assignment of *ROI*_wp_ to PZ vs TZ is made by using variable fraction (10, 15, and 20%) of *ROI*_wp_ in PZ. *ROI*_wp_ in PZ and TZ were denoted *pzROI*_wp_ and *tzROI*_wp_, respectively (Figure 1E). The volumes in PZ outside of *pzROI*_wp_ were considered normal appearing tissues PZ (NAT_PZ_) and TZ (NAT_TZ_).
- *Quantitative feature extraction*. The signal-vs-time curve *S*_ROI_ is reconstructed using Eq. 1 for pixels within *pzROI*_wp_ or *tzROI*_wp_ using *k* = 3; *S*_ROI_ is an approximation of the average of the curves within the region. By using the NMF reconstruction this signal is effectively de-noised (19). *S*_ROI_ and the average curve from the muscle volume *S*_GM_ were used for quantitative analysis (Figure 1F). Six quantitative features (25, 26), summarized in Table 1 and illustrated in Figure S1 in Supplementary Material, were computed based on these two signals (Figure 1G). The features are divided in three groups: (i) Features extracted directly from the DCE-MRI intensity curve; (ii) Features based on the fitting of *S*_ROI_ and *S*_GM_ to a bi-exponential model; and (iii) Features calculated by fitting these curves to a late enhancement linear model. The parameters from the bi-exponential model are: initial static intensity *s*_0_, plateau intensity *s*_m_, start of enhancement *t*_0_, time-to-peak τ, and washout slope *wo*_biexp_ (Figure S1 in Supplementary Material). The wash-in slope *wi* was computed as (*s_m_* *− s_0_*)*/*τ. The late enhancement linear model computed the slope and intercept of a linear fit of the intensities between *DCE_270_* and *DCE_330_*. This slope is referred to as *wo*_linear_.
- *Map of aggressiveness*. Let *F* be the value of a quantitative feature for the curve *S*_ROI_ in the identified volume *ROI*_wp_. Using the distribution of *F* in associations with GS, a spatial map of tumor aggressiveness was computed (Figure 1H). The 3D map of the well-perfused pattern, *W*_wp_, is used to generalize *F* for all pixels in the prostate. Let *W_wp_^i^* be the value of *W*_wp_ in the *i*^th^ pixel of the prostate [Eq. (1)]. Let ω = mean (*W_wp_^i^*, *i* ∈ *ROI*_wp_), i.e., ω is the average of *W*_wp_ in the tumor volume *ROI*_wp_. Thus *W_wp_^i^* F/ω = F, *i* ∈ *ROI*_wp_. Consequently, the value *W_wp_^i^* in each pixel can be scaled by F/ω to generate a map of aggressiveness based on feature F and generalized to the whole prostate.

**Table 1:** List of DCE quantitative features.

	Feature name^a^	Definition	Referenced feature
**Direct from DCE-MRI intensity curve**			

1	Early AUC	Early AUC (*S_ROI_*)	*Early AUC*(*S*) = *AUC*^a^*_60–120_*(*S*)
2	Late AUC	Late AUC (*S_ROI_*)	*Late AUC*(*S*) = *AUC_240–330_*(*S*)

**Bi-exponential model (19)**			

3	Wash-in	Wash-in (*S_ROI_*)	*Wash-in*(*S*) = *wi*(*S*)
4	Early AUFC	Early AUFC (*S_ROI_*)	*Early AUFC*(*S*) = *AUFC_60–120_^b^*(*S*)
5	Late AUFC	Late AUFC (*S_ROI_*)	*Late AUFC*(*S*) = *AUFC_240–330_*(*S*)

**Late enhancement linear model (26)**			

6	Wash-out	*-*[*wash-out*(*S_ROI_*)]	*wash-out*(*S*) = *wo_linear_*(*S*)

*DCE, dynamic contrast enhanced; AUC, area under the curve; AUFC, area under the *fitted* curve estimated using Huisman’s (25) bi-exponential model*.

*Notations: *S*_ROI_ is an approximation of the average of the curves within the well-perfused region reconstructed using NMF (non-negative matrix factorization)*.

*^a^AUC_t1–t2_(*S*) is the area under the curve S between times *t1* and t2*.

*^b^AUFC_t1–t2_(*S*) is the area under the fitted curve S between times *t1* and t2*.

For convenience, the feature map is represented using a 1 to 10 scale, grouping the *W_wp_^i^* *F*/ω in 10 bins. Feature range was bound by 5th and 95th percentile of the feature values in the entire population of subjects with positive biopsies. DCE-feature scores are allocated by uniformly dividing the bounded interval in ten regions. Pixels with feature values smaller than the lower bound or larger than the upper bound were assigned a DCE-feature-score of 1 and 10, respectively.

### Statistical Analysis

Spearman correlation coefficient (ρ) of the DCE-scores with the highest GS from the biopsy session was computed (patient level analysis). The GS were grouped in a clinically relevant categories, separating GS7 (3 + 4) and GS7 (4 + 3) (27). To study the ability of the features to discriminate between *indolent* (GS = 6) and *aggressive* (GS ≥ 7) lesions, the association between the quantitative features and these two classes was investigated. The significance of the median difference between groups was evaluated using Kruskal–Wallis test, followed by Dunn’s posttest. In addition, the AUC of the receiver operating characteristics (ROC) analysis was computed. A subset of the patients underwent MRI-ultrasound fused targeted (MRI-US) biopsies. The statistical test above were carried out for the DCE-scores of each biopsy location and biopsy GS (biopsy level analysis). Statistical analysis was performed using MATLAB. All tests were two-sided. Significance was set at a *p*-value <0.05.

## Results

### Patients

The flow diagram of inclusion and exclusion criteria of the analyzed patients is presented in Figure S2 in Supplementary Material. Nine patients (9/71, 12%) were excluded because: four patients lacked complete DCE-MRI series and five had suboptimal contrast data due to acquisition artifacts or incomplete contrast administration. The clinical characteristics of the analyzed cohort are summarized in Table S1 in Supplementary Material. The highest GS from the biopsy session was used.

### Motion Correction

For each pixel in the dataset, *DCE_N_* was aligned to *DCE_N − 30_*, (*N* = 90, 120, …, 330) and motion was quantified as the mean absolute pixel gray scale-intensity difference between *DCE_N_* and *DCE_N − 30_*, before and after correction. For 27 patients (44%), the movement correction procedure resulted in essentially no change (change of pixels squared differences before and after: median = 0.02%; range = −2.38 to 1.51%). For 35 patients (56%), the procedure yielded significant improvement: median = 9.93%, range = 2.61–46.84%. The procedure is illustrated for the patient with the largest correction (Figure S3 in Supplementary Material).

### Identification and Delineation of Well-Perfused (Suspicious) ROI in the Prostate

**Figure 2:**
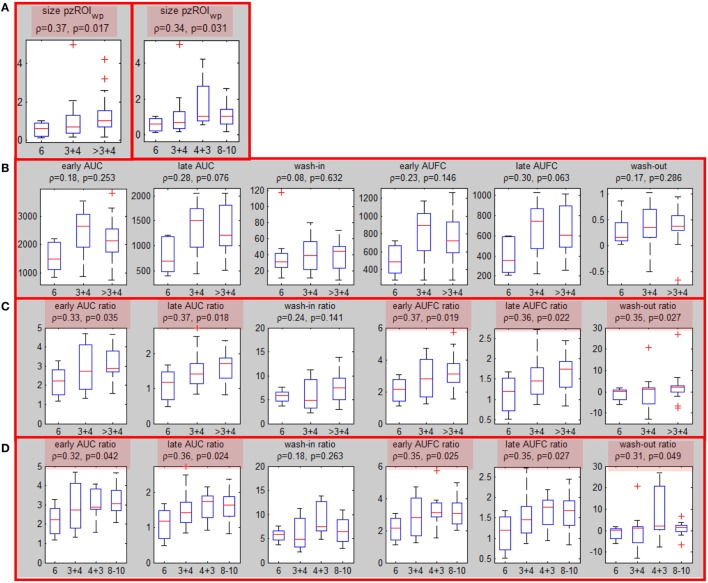
Associations of well-perfused region of interest (*ROI*_wp_) volume and quantitative DCE features with Gleason Score. **(A)** Associations (Spearman correlation coefficient and *p*-values) between peripheral zone *ROI*_wp_ and GS divided in 3 (left) and 4 (right) groups; **(B)** association of the six quantitative DCE features, calculated from the signal-vs-time curve *S*_ROI_ of *pzROI*_wp_ and GS; **(C)** The same as in **(B)** but after normalization with the signal-vs-time from the muscle reference; significant correlations are highlighted in red. **(D)** The same as in **(C)**, but four groups for GS were considered.

All five segmentation methods resulted in significant correlations between the volumes of *ROI*_wp_ and GS. The Spearman correlation ρ of the volume of *ROI*_wp_ and GS (6, 3 + 4, 4 + 3, ≥8), its *p*-value, and the number of patients assigned to PZ or TZ, or rejected due to volumes less than 0.5 cc are presented in Table 2. While in general, with increasing β, the correlations with GS increased, the resulting smaller ROI_wp_ volumes caused a large number of patients to be rejected due to *ROI*_wp_ < 0.5 cc. β = 60 and 10% PZ vs TZ were selected. The median size of the well-perfused region *ROI*_wp_ across the patients was 1.95 cc (range 0.23–12.21 cc). Following 0.05 cc cleanup for disconnected small volumes, the resulting volumes *ROI*_wp_ were on average 18.19% smaller and ranged in size between 0.16 and 11.52 cc. Eight patients with *ROI*_wp_ <0.5 cc were eliminated from further processing. For the remaining 54 patients, the mean volume of *ROI*_wp_ was 2.57 cc (median = 1.84 cc, range 0.51–11.52 cc). Forty-one (76%) of the tumors were in the PZ (*pzROI*_wp_*)*. The mean volume of *pzROI*_wp_ was 1.08 cc (median = 0.80 cc, range 0.15–5.01 cc) and the mean volume of *tzROI*_wp_ was 3.09 cc (median = 1.28 cc, range 0.80–11.52 cc). The distributions of *pzROI*_wp_ volumes in association with GS divided in three (6, 3 + 4, >4 + 3) and four groups (6, 3 + 4, 4 + 3, 8 − 10) are shown in Figure 2A. In both cases, Spearman correlation coefficients were significant.

**Table 2:** Associations between Gleason Score and volumes of automatically segmented suspicious regions of interest (ROIs).

PZ^a^	Statistics	Otsu	40%	50%	60%	70%
10%	Spearman’s ρ	0.22	0.14	0.25	0.34	0.43
	*p*-value	0.155	0.307	0.087	0.031	0.086
	PZ/TZ/rejected (*N*)	45/15/0	53/7/0	48/9/3	40/12/8	17/11/32

15%	Spearman’s ρ	0.19	0.13	0.27	0.39	0.43
	*p*-value	0.230	0.364	0.096	0.022	0.086
	PZ/TZ/rejected (*N*)	40/20/0	51/9/0	40/17/3	35/17/8	17/11/32

20%	Spearman’s ρ	0.14	0.14	0.23	0.33	0.21
	*p*-value	0.420	0.353	0.166	0.073	0.443
	PZ/TZ/rejected (*N*)	34/26/0	46/14/0	38/19/3	31/21/8	15/13/32

*PZ, peripheral zone; TZ, transition zone*.

*^a^Threshold (%) of the suspicious ROIs in PZ*.

### Associations of Quantitative Features with Prostate Cancer Aggressiveness (Patient Level Analysis)

Six quantitative features (Table 1) were computed based on the signal *S*_ROI_ for the patients with suspicious area in PZ (*pzROI*_wp_*)*. The correlations between these features and the highest GS (6, 3 + 4 and >3 + 4) are presented before and after normalization with the signal from the muscle reference *S*_GM_ (Figures 2B,C). Note that the correlations with GS improve substantially following normalization with the muscle signal, reaching significance (*p* < 0.05) in all cases but one. When GS is divided in four groups: 6, 3 + 4, 4 + 3, 8 − 10 the same five features remained significant (Figure 2D). It should be noted that none of the features were significant for *tzROI_wp_* (data not shown). In addition, comparisons including the distribution of the quantitative features in NAT_PZ_ and NAT_TZ_ we carried out (Figure S4 in Supplementary Material). Their corresponding AUC for discrimination between NAT_PZ_ and *pzROI_wp_* ranged between 0.62 and 0.99. AUCs of DCE-scores in *tzROI_wp_* were also high. In view of the small number of lesions in TZ, however, these results should be treated with caution.

### Correlation of Quantitative Features with MRI-US Biopsies (Biopsy Level Analysis)

**Figure 3:**
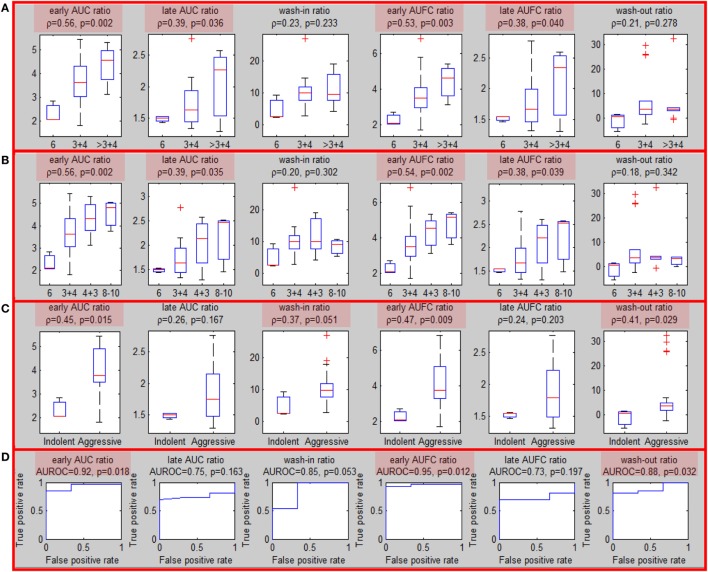
Association of quantitative DCE features and Gleason Score for MRI-US biopsies. **(A–C)** Associations of quantitative DCE features in MRI-US well-perfused ROI (*usROI*_wp_) with GS, separated in three, four, and two groups [indolent (GS = 6) vs aggressive (GS > 6)]. Significant associations are highlighted in red; **(D)** area under the curve for discrimination between indolent and aggressive tumors.

Sixteen patients underwent MRI-US biopsies and had total of 35 positive biopsies (Figure S5 in Supplementary Material). After confirmation of the location of the actual biopsy, the region with needle tracks was back-projected onto T2w. The overlap between the biopsy region and the well-perfused region *ROI*_wp_ was denoted as *usROI*_wp_. Quantitative features were computed for each biopsy in PZ (29/35) using the average signal in *usROI*_wp_. The associations of the sixfeatures for PZ tumors and the targeted biopsy GS (divided in three and four groups) are presented in Figures 3A,B. The same four features were significant using three, four GS groups and when tumors were split into indolent (GS = 6) and aggressive (GS ≥ 7) [*wash-in* ratio reached marginal (*p* = 0.051) significance] (Figure 3C). The features resulted in AUC for classifying indolent vs aggressive PZ lesions ranging between 0.85 and 0.95 (Figure 3D).

### Map of Aggressiveness

**Figure 4:**
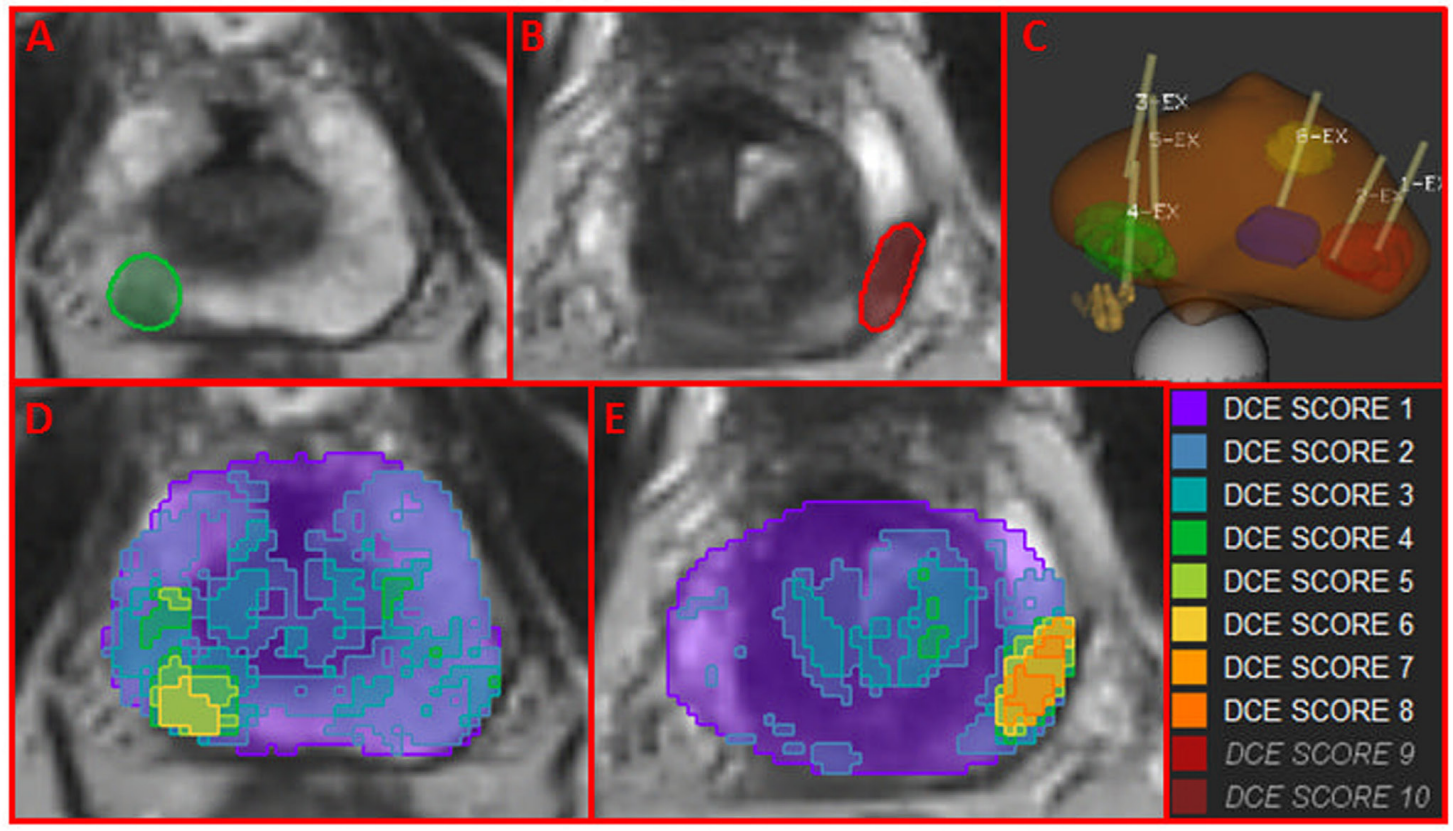
Map of aggressiveness, using location and Gleason Score of MRI-US biopsies. **(A,B)** Axial slices of the prostate with mapped regions (in green and red) of the intersection of the identified well-perfused region *ROI*_wp_ and the biopsy locations; **(C)** schematic representation of targeted biopsies with the biopsy needle tracks (yellow) in the target volumes. On pathology review, the biopsies were assigned GS = 6 (green target) and GS = 7 (for both biopsies in the red target). The blue target biopsy in transition zone was found benign; **(D,E)** map of aggressiveness, generated for *earlyAUFCrat* feature overlaid on the MRIs in **(A,B)**. Color scheme of maps is given on the right. The areas of the positive biopsies are clearly identified.

Using *W*_wp_ and the DCE-score F from *ROI*_wp_, each pixel in the prostate was scored for aggressiveness using 1 to 10 scale. In Figures 4A,B, two axial slices of the prostate are shown with indicated regions representing the intersection of the identified well-perfused region *ROI*_wp_ and the biopsy locations. A schematic representation of the prostate with the biopsy needle tracks (yellow) in the target volumes (1 in the green, 2 in the red, and 1 in the blue target) is depicted in Figure 4C. On pathology review, the biopsies were assigned GS = 6 (green target) and GS = 7 (for both biopsies in the red target). Map of aggressiveness was generated for *early AUFC ratio* feature. In Figures 4D,E, the map is overlaid on the patient axial MRIs slices. The areas of the positive biopsies are clearly identified. The color scheme of the maps is given on the right. There are no pixels with DCE-scores 9 and 10, indicating lack of areas with high GS. The highest DCE-score on the maps (left to right) was 6 and 8, consistent with the biopsy findings.

The MRI-US dataset was utilized to confirm the accuracy of the global map of aggressiveness. On one hand, the map of aggressiveness *feature_Wwp_* was generated using only *W*_wp_ and *F*. On the other hand, the feature value for each biopsy ROI was estimated in the previous section using *usROI*_wp_. In Figure S6 in Supplementary Material, the *x*-axis corresponds to the feature computed using *usROI_wp_*. The *y*-axis is the mean value of the map of aggressiveness over the region of the biopsy, mean [*feature_Wwp_*(*mrusROI*)]. There was an excellent concordance between these two estimates for each biopsy ROI (*n* = 29) for each feature: the correlation coefficients ranged between 0.85 and 0.99; the slope for the linear fit ranged between 0.87 and 0.98. This graph also confirms the co-localization of the “hot” spots of the map of aggressiveness with the biopsy location.

## Discussion

DCE-MRI plays an ancillary role in tumor assessment to T2-weighted (T2w) and DWI in the contemporary recommendations for prostate cancer review and scoring in PI-RADSv2. The diminished role of DCE-MRI is confounded by: (i) difficulties for radiologists to quantitatively assess multiple DCE-MRI series via image viewing; and (ii) lack of reproducibility of the quantitative parameters when modeling approaches are used. In addition, other factors, such as chronic inflammation and differential characteristics of the prostate zones further complicate the DCE-MRI interpretation. The thesis of this work is that DCE-MRI can reliably identify and consistently score prostate cancer lesions if the region of well-perfused tissue is properly delineated and sensible quantitative features, using internal signal normalization are used.

The presented approach considers the signal-vs-time curves from each pixel in the dataset and an unsupervised PR technique is used to identify the pixels with the characteristic for tumor perfusion pattern. Quantitative features are calculated from a representative signal-vs-time curve from these pixels. Several of these features, especially after normalization with signal from the muscle, were significantly correlated with biopsy GS, and differentiated indolent (GS = 6) from aggressive tumors (GS ≥ 7). For instance, Litjens et al. (28) found that the τ-induced volume and *wash-out* had a large relative importance in their ROC analysis for classification of cancer (normal/benign). Vos et al. (10) found that *wash-in* and *wash-out* were statistically different between low-grade (GS = 6) and high-grade [GS > 7(4 + 3)] in the PZ. The Area under the Receiver Operating Characteristics Curve (AUROC) for indolent vs. aggressive in this work were 0.72 for wash-in and 0.65 for wash-out. In comparison, after normalization by muscle, the AUROC for wash-in and wash-out of 0.85 and 0.88 are reported here. Chen et al. found a significant correlation between wash-out and GS, with an AUROC for indolent/aggressive classification of 0.88 (26). Significant correlation and coincidentally the same AUROC of 0.88 is reported here. It should be noted that unlike these previous reports, where few significant features are found, the majority of the evaluated features here were significantly correlated with GS, both on patient and biopsy level analysis. This is attributed to the robustness of well-perfused ROI selection and fitting a single DCE curve of high SNR, rather than pixel-by-pixel in the ROI (10). This reduces the errors associated with noise, data acquisition and motion artifacts.

Here, a novel pipeline for automatic generation of a well-perfused ROI is presented. The method performs well in datasets acquired at low temporal resolution. Most current research and PI-RADS guidelines suggest that high-temporal resolution be used. While this is a requirement for compartment model analysis, the straight-forward features extracted here are significantly correlated with GS even at low temporal resolution (Δt = 30 s).

This study has several limitations. First, this is a retrospective study with all inherent limitations of the design. Second, the application of the thresholds require contours of PZ and TZ. The use of prostate atlas will decrease significantly the need of manual contouring (29). And finally, the generalizability of the determined thresholds to other MRI sequences, vendors, magnetic field strengths, and coils (endorectal vs body) should be investigated. Until then, it should be assumed that the thresholds are valid for DCE data, acquired under identical conditions.

In conclusion, a quantitative approach for DCE-MRI analysis is developed and the resultant map of aggressiveness is integrated in radiotherapy/radiology imaging software as a guide for targeted biopsies and treatments. In RT of prostate cancer, dose escalation for prostate cancer has been shown to reduce biochemical failure (30). While dose escalation also has been shown to reduce the need for androgen deprivation in intermediate to high risk patients (4, 31, 32), when the entire prostate is dose escalated, there is an increased risk of secondary adverse events. Dose escalation only to determinate prostate habitats has the potential to improve tumor control with less toxicity than when the entire prostate is dose escalated.

## Ethics Statement

This study was carried out in accordance with the recommendations of the Institutional Review Board (IRB) of the University of Miami. The IRB approved a protocol for retrospective review of mpMRI exams from prostate cancer patients. The IRB waived the need for informed consent.

## Author Contributions

RS, AP, and NP conceived the study; AP, MA, and SP provided patients; NP, RS, FC, and OK collected and assembled data; NP, RS, BM, FM, RC, and AP conducted data analysis and interpretation; NP, RS, and AP wrote the manuscript; All authors reviewed the manuscript for intellectual content and approved the final version.

## Conflict of Interest Statement

The authors declare that the research was conducted in the absence of any commercial or financial relationships that could be construed as a potential conflict of interest.
