# Supplementary figures and images for: Automatic Detection and Quantitative DCE-MRI Scoring of Prostate Cancer Aggressiveness
Source: Front Oncol. 2017 Nov 10;7:259. doi: 10.3389/fonc.2017.00259 (PMC5686056; doi:10.3389/fonc.2017.00259)

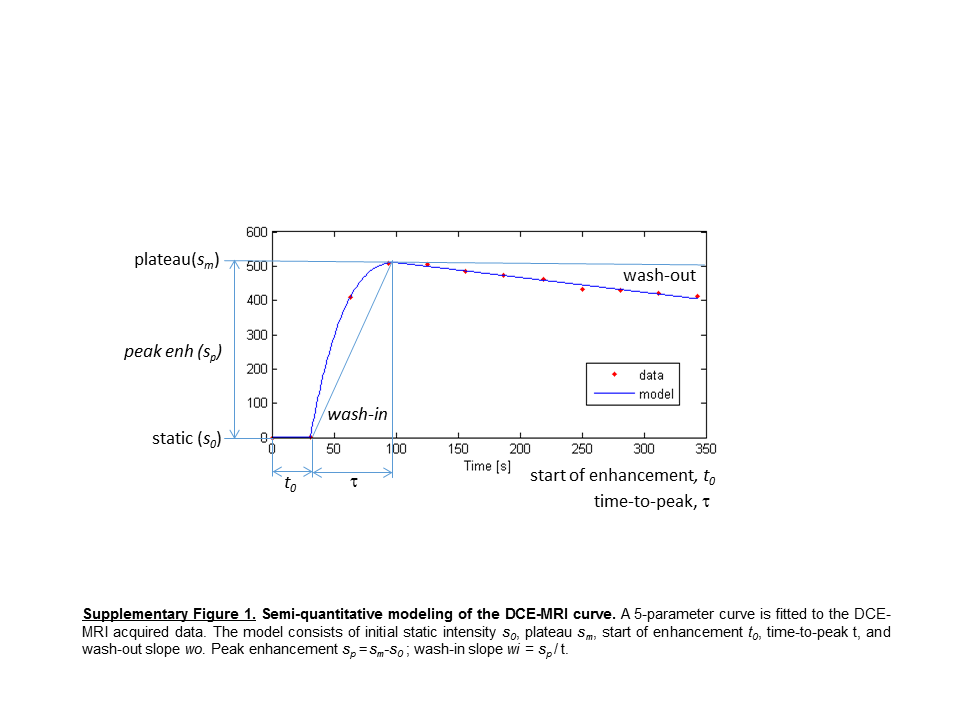

Supplement: Supplementary file 1 [file image_1.tif]

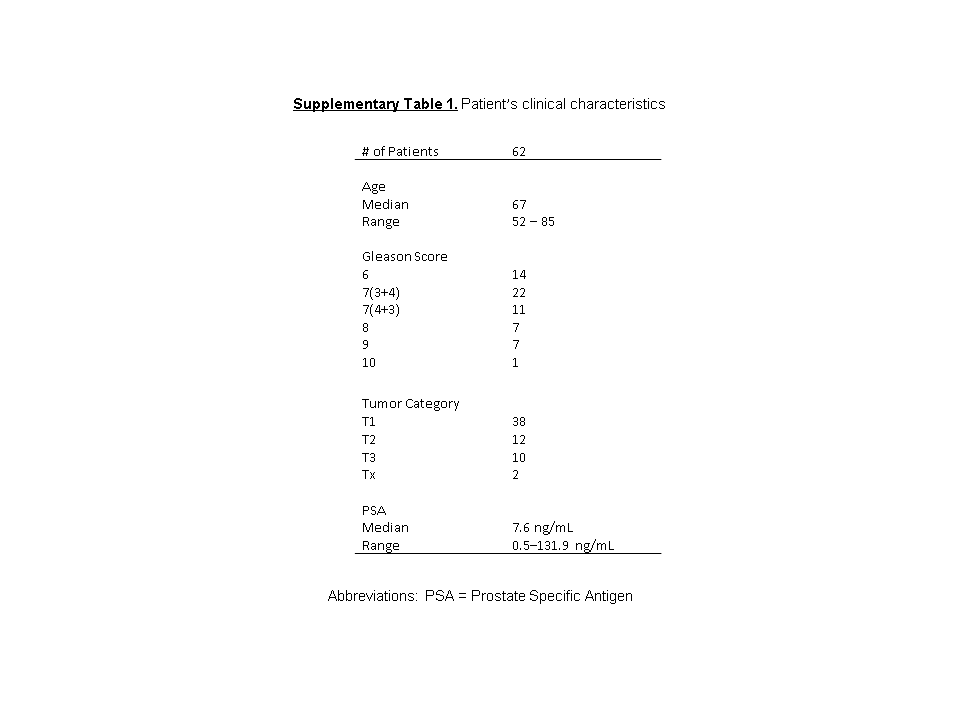

Supplement: Supplementary file 2 [file image_2.tif]

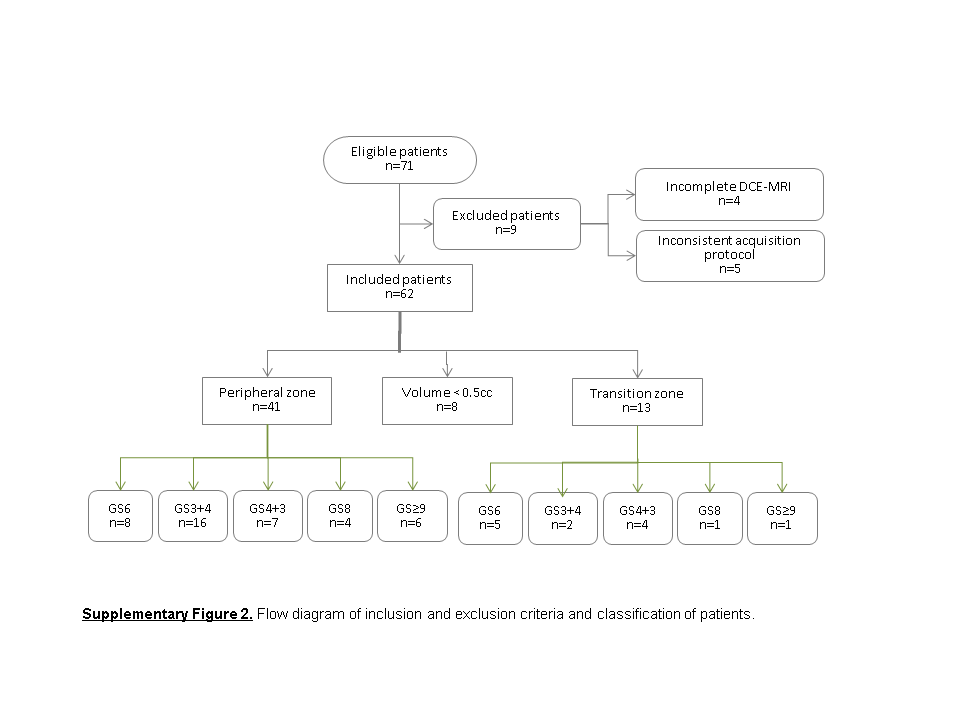

Supplement: Supplementary file 3 [file image_3.tif]

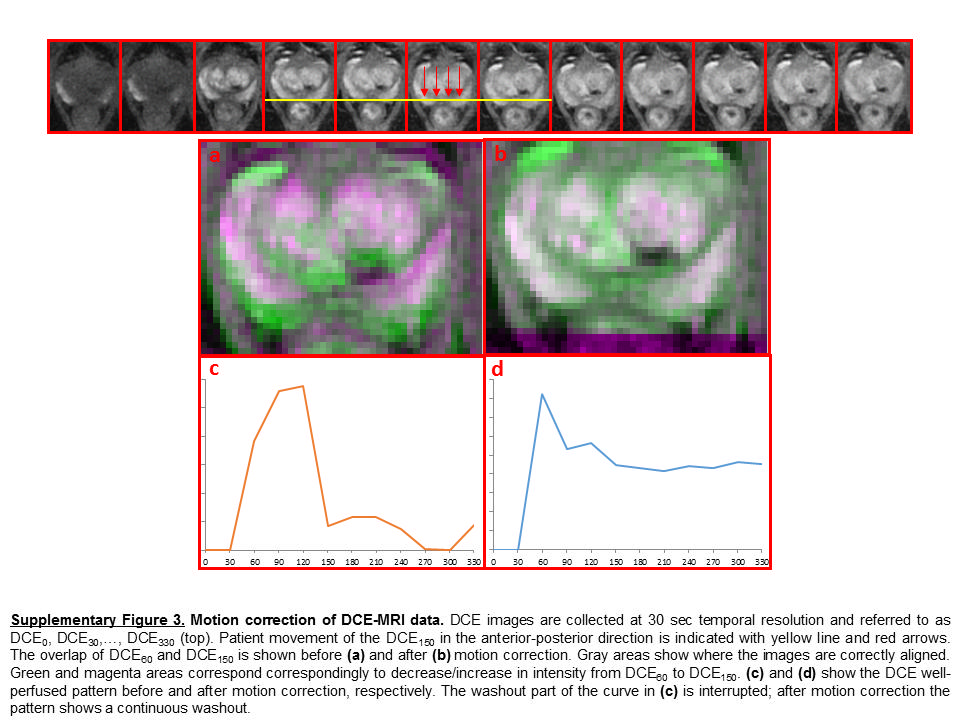

Supplement: Supplementary file 4 [file image_4.tif]

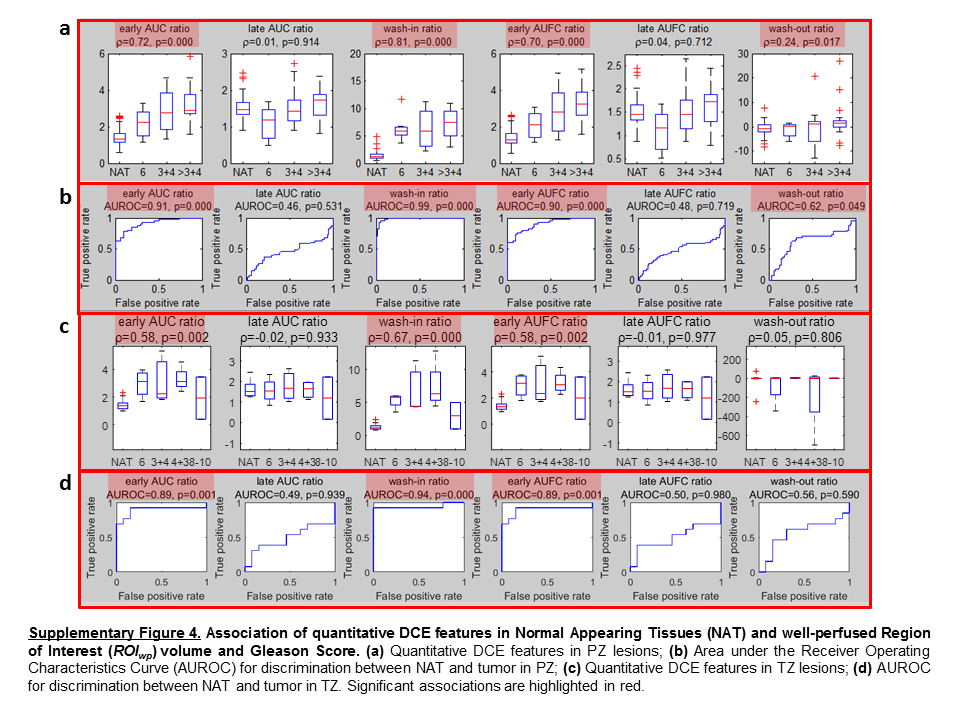

Supplement: Supplementary file 5 [file image_5.tif]

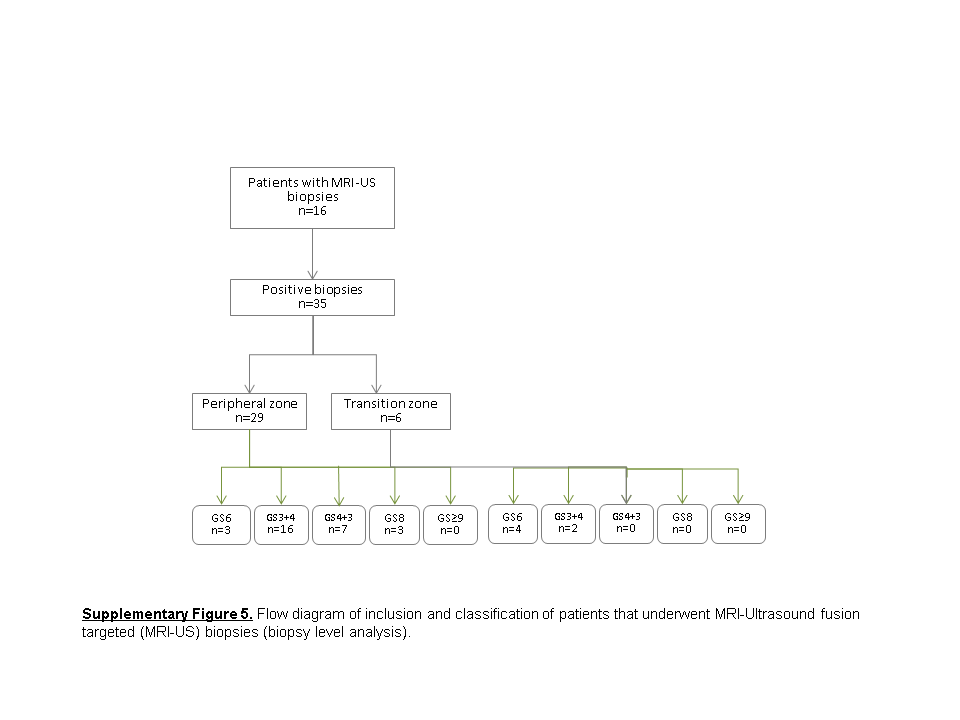

Supplement: Supplementary file 6 [file image_6.tif]

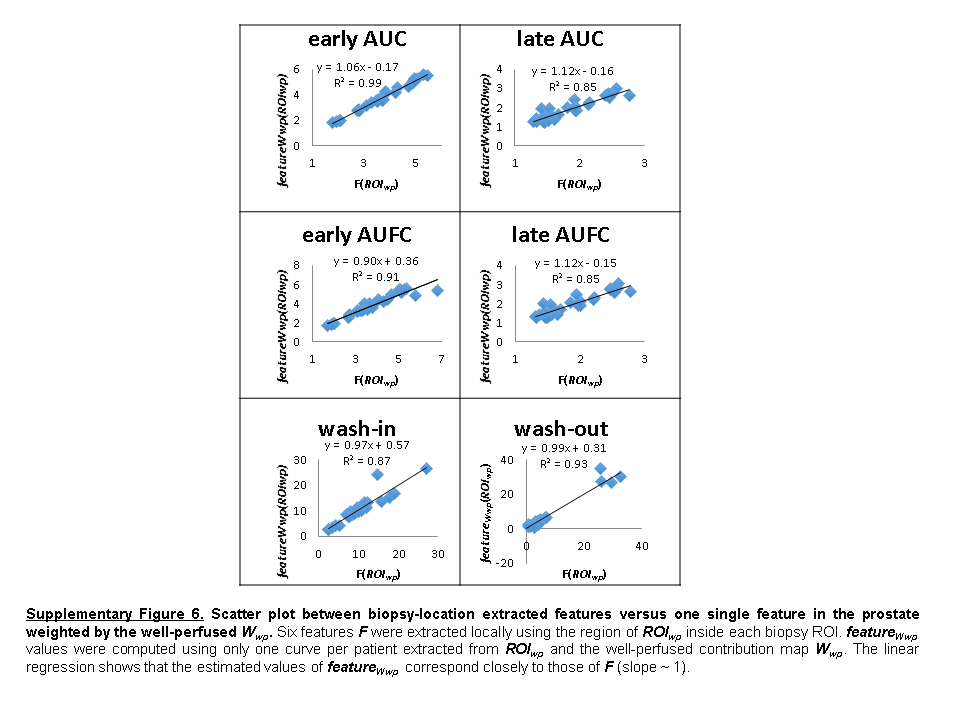

Supplement: Supplementary file 7 [file image_7.tif]
